# Comparison of the Performance of Three Blood Culture Systems in a Chinese Tertiary-Care Hospital

**DOI:** 10.3389/fcimb.2019.00285

**Published:** 2019-08-07

**Authors:** Guanlin Li, Jingjing Sun, Shoucheng Pan, Wenti Li, Shijie Zhang, Yongfeng Wang, Xiaoxu Sun, Hui Xu, Liang Ming

**Affiliations:** Department of Clinical Laboratory, First Affiliated Hospital of Zhengzhou University, Zhengzhou, China

**Keywords:** BACTEC Plus, BacT/Alert, VersaTREK, blood culture system, simulated blood culture, bloodstream infection

## Abstract

In this study, we evaluated the performance of three blood culture systems in a Chinese tertiary-care hospital. Samples of simulated bacteremia were prepared using 10 mL of fresh blood from healthy humans and bacterial suspensions of known cell density. Portions of the specimens were treated with an antibiotic or antifungal drug at specified concentrations to simulate antibacterial drug treatment. We analyzed three blood culture systems: BACTEC Plus, BacT/Alert, and VersaTREK. Both time-to-detection (TTD) of 10 types of bacteria and five types of yeasts in samples without antibiotic treatment and positive detection rate of samples treated with different concentrations of antibiotic or antifungal drugs were compared among the culture systems. We also retrospectively analyzed the use of the culture systems in our hospital from 2015 to 2018. In the simulated study, in the absence of antibiotics, the VersaTREK REDOX 1 displayed the shortest TTD for *Pseudomonas aeruginosa, Haemophilus influenzae, Staphylococcus aureus, Streptococcus pneumoniae, Candida albicans*, and *Candida glabrata* (*P* < 0.001). Among the anaerobically cultured samples, BACTEC lytic/10 anaerobic/F displayed the shortest TTD for *Escherichia coli, S. aureus, Enterococcus faecalis, S. pneumoniae, Bacteroides fragilis*, and *Bacteroides vulgatus* (*P* < 0.001). Comparatively, BacT/Alert FA/FN showed no advantages. In antibiotic-treated samples, overall recovery rates for the BACTEC, BacT/Alert, and VersaTREK systems were, were 70.2, 43.7, and 27.4%, respectively. BACTEC facilitated higher recovery rate than the other two systems (*P* < 0.001). In antifungal treatment, the overall recovery rates for the BACTEC, BacT/Alert, and VersaTREK systems were 93.9, 98.3, and 69.4%, respectively. BACTEC Plus showed a recovery rate comparable to that of BacT/Alert (*P* = 0.835), and the recovery rate of both these systems was higher than that of VersaTREK (*P* < 0.001). The TTD values and positive rates determined in the retrospective study were consistent with those obtained in the simulated study. The combination of BACTEC PLUS Aerobic/F and BACTEC lytic/10 anaerobic/F culture systems displayed the best clinical performance. Furthermore, the BacT/Alert FAN culture system was found to be more resistant to antifungal drugs and levofloxacin, whereas the VersaTREK system is considered more suitable for primary blood cultures without antibiotic supplementation.

## Introduction

Systemic infections are a prominent cause of morbidity and mortality worldwide and influence the duration of hospitalization and associated healthcare costs (Tumbarello et al., 2007; Goto and Al-Hasan, 2013). Accordingly, the accurate and timely detection of bacteremia is important for determining the appropriate treatment method for systemic sepsis (Barenfanger et al., 2008; Funke and Kumar, 2011). Currently, three automated microbial detection systems have been approved for use in the detection of bacteremia and fungemia, namely, the BacT/Alert 3D (3D) (bioMerieux, Durham, NC), BD Bactec Plus (BD Diagnostics, Sparks, MD), and VersaTREK (TREK Diagnostic Systems, Cleveland, OH) systems. The BacT/Alert automated microbial detection system uses an internal colorimetric sensor that changes from gray to yellow in the presence of CO_2_ produced by growing microorganisms and utilizes active carbon to remove antibiotics from blood samples. The BD Bactec Plus is based on a chemical sensor that can detect increases in CO_2_ produced as a consequence of microbial growth. The sensor in this system is monitored at 10-min intervals for an increase in fluorescence, which is proportional to the amount of CO_2_ present, and the system uses cationic-exchange and adsorbent nonionic resins to remove antibiotics from blood samples. The VersaTREK automated microbial detection system, which evolved from and replaced the Difco ESP culture II system, was introduced commercially in 2003. The detection of positive cultures using this system is based on the measurement of pressure changes in the sample bottle headspace (due to consumption and production of gases by microorganisms) using an external pressure sensor, and relies on an optimal 1:9 blood:broth dilution to neutralize the effects of antibiotics.

Differences in the performance of these three blood culture systems have been reported previously (Horvath et al., 2003, 2004, 2007; Søgaard et al., 2006; Flayhart et al., 2007; Mirrett et al., 2007; Miller et al., 2011; Riedel et al., 2011). In the present study, we simultaneously evaluated the performance of these three blood culture systems, using samples obtained via simulated bacterial inoculation, and directly compared the ability of each system to neutralize various antibiotics that are currently used extensively in Chinese hospitals at simulated trough (T), mid-level (M), and peak (P) therapeutic concentrations in serum challenged with potential systemic pathogens. Moreover, we compared the potential of these three systems to detect *Candida* spp. in seeded blood culture specimens in the presence or absence of therapeutic levels of antifungal agents. Compared with previous studies, we analyzed an expanded range of pathogens and antibiotics or antifungals in the present study. Our results may contribute to explaining the positive detection rate for blood cultures in our hospital from 2015 to 2018, and also changes in the trends of antibiotic resistance among members of family *Enterobacteriaceae* isolated from blood cultures in the past 4 years.

## Materials and Methods

### Study Setting

The study was conducted at the First Affiliated Hospital of Zhengzhou University, which contains 10,000 beds, 236 wards, five intensive care units, and an emergency center. In 2018, the number of outpatient visits to the hospital exceeded 6.89 million.

Approval for this study was obtained from the ethics board of the First Affiliated Hospital of Zhengzhou University, as was informed consent from their legal representative.

### Media

In this study, we used the following commonly used commercial blood culture media: BACTEC PLUS Aerobic/F and BACTEC lytic/10 anaerobic/F (BD Diagnostics, Sparks, MD, USA), BacT/Alert FA and BacT/Alert FN (bioMerieux, Durham, NC, USA), and VersaTREK REDOX 1 and REDOX2 (80 mL; TREK Diagnostic Systems, Cleveland, OH, USA). Each type of bottle used in the analyses was from the same batch. The human blood used in the study was purchased from the Red Cross Blood Center of Henan Province and was drawn not more than 5 days prior to use and was stored at 4°C.

### Antibiotic Agents

The following antibiotic drugs were used in the present study: ampicillin (3, 12, and 47 μg/mL); levofloxacin (Daiichi Sankyo:13, 45, and 120 μg/mL); azithromycin (Zithromax, Pfizer: 0.2, 0.4, and 3.63 μg/mL); vancomycin (Lilly: 10, 25, and 50 μg/mL); ceftazidime (GSK: 1.16, 20, and 130 μg/mL); an cefoperazone/sulbactam (sulperazon: 11/5.5, 43/21.5, and 105/52.5 μg/mL). These antibiotics were diluted with deionized water to the indicated final concentrations, which corresponded to trough, mid-, and peak therapeutic serum levels (Amsden, 2000).

### Antifungal Agents

All isolates were tested against the following commonly used antifungal agents at the indicated trough- and peak-level concentrations (Andes et al., 2009): voriconazole (Vfend, Pfizer: 3.06 and 4.7 μg/mL); fluconazole (diflucan; Pfizer: 4.18 and 6.72 μg/mL); amphotericin B (0.5 and 2 μg/mL); caspofungin (Merck: 1.6 and 8 μg/mL); and itraconazole (Sporanox; Janssen/Janssen-Cilag, Belgium: 0.523 and 3.021 μg/mL).

### Organisms

As test organisms for the present study, we used the following ATCC strains: *Staphylococcus aureus* ATCC 25923, *Streptococcus pneumoniae* ATCC 49619, *Escherichia coli* ATCC 25922, *Haemophilus influenzae* ATCC 49247, *Pseudomonas aeruginosa* ATCC 27853, *Enterococcus faecalis* ATCC29212, *Bacteroides fragilis* ATCC 25285, *Propionibacterium acnes* ATCC 11827, *Prevotella melaninogenica* ATCC 25845, *Candida albicans* ATCC 10231, *Candida. glabrata* ATCC 2001, *Candida tropicalis* ATCC 750, *Candida krusei* ATCC 6258, and *Candida parapsilosis* ATCC 22019. *Brucella melitensis, Clostridium ramosum, Bacteroides vulgatus*, and methicillin-resistant *S. aureus* were isolated from unique clinical specimens and identified using VITEK compact and VITEK MS systems (bioMérieux). All strains were stored at −80°C and used after three rounds of sub-culturing to ensure the viability of the strains. These strains were resuspended in 0.9% saline to yield a 0.5 McFarland bacterial suspension. Suspensions of 10–100 CFU/mL were subsequently obtained via a series of 100-fold dilutions.

### Bottle Inoculation and Incubation

Each drug/organism combination comprised 10 mL of human whole blood, 0.5 mL of bacterial suspension, and 0.1 mL of antimicrobial substance. The positive control comprised 10 mL of human whole blood and 0.5 mL of the bacterial suspension without an antimicrobial drug, whereas the negative control comprised 10 mL human blood and 0.6 mL of saline. Each set of bottles contained a growth control, and for each test organism, we examined the effects of three concentrations of antibiotics in triplicate analyses. All bottles were incubated for 5 days using the dedicated system equipment. Analyses of drug concentration/organism combinations were repeated three times, and experiments were performed twice on two different dates. When cultures showed a positive result, time-to-detection (TTD) values were recorded, followed by smearing, staining, and sub-culturing.

### Determination of Voriconazole Concentration via High-Performance Liquid Chromatography (HPLC)

In order to compare the adsorption and/or dilution capacity of the three blood culture systems against fungal agents, sample bottles were inoculated with 10 mL of banked blood and various concentrations of voriconazole. A 2 mL aliquot of the blood was immediately removed and centrifuged at 1,400 rpm for 10 min. The bottles were then loaded into the system instruments and incubated for 1 h, after which time an additional 2 mL was removed and centrifuged at 1,400 rpm for 10 min. Residual concentrations of voriconazole in culture media at 0 and 1 h after drug supplementation were determined via HPLC, as reported previously (Zhang et al., 2013). Experiments were performed in triplicate.

### Retrospective Evaluation of Blood Culture Systems

We retrospectively obtained data regarding TTD using the three evaluated blood culture systems. During the period from January 2015 to December 2018, BACTEC blood culture systems were used in our hospital from January 2015 to December 2015, whereas the BacT/Alert system has been used continuously since 2016, and the VersaTREK culture system was used from January 2017 to December 2018. For each patient, we analyzed a set of two blood cultures, comprising one bottle of aerobic, and one bottle of anaerobic culture. Samples obtained from children under the age of 15 years old, for whom a child bottle was used, or for whom less than two sets of blood culture were available were excluded. Each positive alarm time was recorded to compare the number of alarms for each system at different timepoints. Simultaneously, we analyzed data regarding antimicrobial resistance among members of family *Enterobacteriaceae* isolated from each blood culture system to determine the potential of each system to eliminate antibiotics.

### Statistical Analysis

The ability of each blood culture system to recover microorganisms was evaluated using the Chi-square test. TTD values for positive blood cultures were analyzed using an independent-samples *t*-test. Cultures displaying no microbial growth after 120 h were considered negative. Statistical analysis was performed using SPSS 19.0 (IBM), and a *P* < 0.05 was considered indicative of a significant difference. The sensitivity of members of the family *Enterobacteriaceae* to each drug was analyzed using WHONET 5.6.

## Results

In this study, we compared the TTD values of common clinical pathogens cultured using three blood culture systems. As shown in Table 1, BACTEC PLUS Aerobic/F displayed a shorter TTD for *E. coli* and *E. faecalis*, whereas VersaTREK REDOX 1 displayed a shorter TTD for *P. aeruginosa, S. aureus*, and *S. pneumoniae*. However, the BacT/Alert FA system was not superior to the other two systems with respect to its TTD for bacterial detection. We detected no significant differences among the three systems with regards to the TTD for *Brucella*. As shown in Table 2, BACTEC lytic/10 anaerobic/F displayed the shortest TTD for *E. coli, S. aureus, E. faecalis, S. pneumoniae, B. fragilis, C. ramosum*, and *B. vulgatus. P. aeruginosa* grew exclusively in the Redox2 system, whereas *H. influenzae* grew exclusively in the BacT/Alert FN system, and *B. melitensis* failed to grow in anaerobic culture systems.

**Table 1:** The recovery rate and the average time to detection (TTD) of different organisms cultured aerobically in the three culture systems.

**Organism**	**BACTEC PLUS Aerobic/F**		**BacT/Alert FA**		**VersaTREK REDOX 1**		***P*1**	***P*2**	***P*3**
	**Recovery**	**Average TTD (hours)**	**Recovery**	**Average TTD (hours)**	**Recovery**	**Average TTD (hours)**			
*E. coli*	6/6 (100)	10.65	6/6 (100)	14.05	6/6 (100)	11.3	<0.001	0.076	<0.001
*P. aeruginosa*	6/6 (100)	16.86	6/6 (100)	19.64	6/6 (100)	14.93	<0.001	0.001	<0.001
*H. influenzae*	6/6 (100)	20.19	6/6 (100)	24.93	6/6 (100)	17.93	<0.001	0.001	<0.001
*S. aureus*	6/6 (100)	15.16	6/6 (100)	16.04	6/6 (100)	11.13	0.015	<0.001	<0.001
*E. faecalis*	6/6 (100)	11.78	6/6 (100)	15.13	6/6 (100)	12.48	<0.001	0.18	<0.001
*S. pneumoniae*	6/6 (100)	18.87	6/6 (100)	18.84	6/6 (100)	16.92	0.94	<0.001	<0.001
*B. melitensis*	6/6 (100)	62.34	6/6 (100)	56.54	6/6 (100)	59.59	0.107	0.429	0.381
*C. albicans*	6/6 (100)	30.58	6/6 (100)	35.20	6/6 (100)	25.82	0.003	0.002	<0.001
*C. kruseii*	6/6 (100)	28.27	6/6 (100)	30.81	6/6 (100)	29.87	0.004	0.053	0.234
*D. parapsilosis*	6/6 (100)	34.51	6/6 (100)	38.00	6/6 (100)	34.48	0.002	0.969	0.002
*C. tropicalis*	6/6 (100)	21.25	6/6 (100)	23.52	6/6 (100)	21.0	0.077	0.839	0.053
*C. glabrata*	6/6 (100)	37.69	6/6 (100)	34.63	6/6 (100)	25.37	0.012	<0.001	<0.001

*“Recovery” denotes the number of bottles from which organisms were recovered presented as a No. (%). TTD values are shown as hours (rounded to the nearest decimal)*.

*P1 values represent comparisons between BACTEC PLUS Aerobic/F and BacT/Alert FA; P2 values represent comparisons between Bactec and VersaTREK REDOX 1; P3 values represent comparisons between BacT/Alert FA vs. VersaTREK REDOX 1*.

**Table 2:** The recovery rate and the average time to detection (TTD) of different organisms cultured anaerobically in three culture systems.

**Organism**	**BACTEC lytic/F**		**BacT/Alert FN**		**VersaTREK REDOX 2**		***P*1**	***P*2**	***P*3**
	**Recovery**	**Average TTD**	**Recover**	**Average TTD**	**Recovery**	**Average TTD**			
*E. coli*	6/6 (100)	9.82	6/6 (100)	11.52	6/6 (100)	13.37	<0.001	<0.001	<0.001
*P. aeruginosa*	0/6 (0)	−	0/6 (0)	−	5/6 (83.3)	24.8	−	−	−
*H. influenzae*	0/6 (0)	−	6/6 (100)	33.29	0/6 (0)	−	−	−	−
*S. aureus*	6/6 (100)	13.47	6/6 (100)	18.58	6/6 (100)	29.35	0.008	<0.001	<0.001
*E. faecalis*	6/6 (100)	12.01	6/6 (100)	14.26	6/6 (100)	16.07	<0.001	<0.001	<0.001
*S. pneumoniae*	6/6 (100)	19.84	6/6 (100)	31.00	6/6 (100)	27.57	<0.001	<0.001	0.009
*B. melitensis*	0/6 (0)	−	0/6 (100)	−	0/6 (0)	−	−	−	−
*B. fragilis*	6/6 (100)	28.13	6/6 (100)	32.25	6/6 (100)	37.42	<0.001	<0.001	<0.001
*C. ramosum*	6/6 (100)	31.37	6/6 (100)	33.30	6/6 (100)	41.56	0.217	0.004	0.017
*B. vulgatus*	6/6 (100)	45.54	6/6 (100)	48.87	6/6 (100)	55.01	0.005	<0.001	<0.001

*“Recovery” denotes the number of bottles from which organisms were recovered presented as No. (%). TTD values are shown as hours (values are rounded to the nearest decimal)*.

*P1 values represent comparisons between BACTEC lytic/F and BacT/Alert FN; P2 values represent comparisons between BACTEC lytic/F and VersaTREK REDOX 2; P3 values represent comparisons between BacT/Alert FN and VersaTREK REDOX 2*.

*“−” indicates no organism growth in any bottles*.

Of the 30 blood culture bottles inoculated with fungi in all systems, the VersaTREK REDOX 1 system displayed the shortest TTD for *Candida* spp., particularly *C. albicans*, and *C. glabrata* (Table 1).

Upon antibiotic supplementation, recovery using the BACTEC, BacT/Alert, and VersaTREK systems was 88.1% (74/84), 57.1% (48/84), and 61.9% (52/84) at the T level; 65.5 (55/84), 45.2% (38/84), and 15.5% (13/84) at the M level; and 57.1% (48/84), 28.6% (24/84), and 4.8% (4/84) at the *P* level, respectively. Overall recoveries were 70.2, 43.7, and 27.4%, respectively (Table 3). Compared with the other two systems, the BACTEC system showed a higher detection rate at all concentration of the antibiotics examined (*P* < 0.05). However, we detected no significant differences in the detection rates of BacT/Alert and VersaTREK at low concentrations of antibiotics (*P* = 0.53).

**Table 3:** Microorganism-specific recovery rate at different antibiotic concentrations.

**Drug (Concn)^a^**	**Organism**	**No. (%) of bottles positive at indicated concentration**								
		**BACTEC PLUS Aerobic/F**			**BacT/Alert FA**			**REDOX 1**		
		**T**	**M**	**P**	**T**	**M**	**P**	**T**	**M**	**P**
Ampicillin (3, 12, 47)	*E. faecalis*	100	100	100	0	0	0	66.7	0	0
	*S. pneumoniae*	100	0	0	0	0	0	0	0	0
Levofloxacin (13, 45, 120)	*S. pneumoniae*	100	100	100	100	100	100	100	0	0
	*E. coli*	0	0	0	100	100	0	0	0	0
	*P. aeruginosa*	66.7	0	0	100	100	100	100	0	0
Azithromycin (0.2, 0.4, 3.63)	*S. pneumoniae*	100	100	100	100	100	100	100	100	0
	*H. influenzae*	100	100	100	100	100	100	100	100	66.7
Vancomycin (10, 25, 50)	*S. aureus MSSA*	100	100	100	0	0	0	0	0	0
	*S. aureus MRSA*	100	100	100	0	0	0	0	0	0
	*E. faecalis*	100	100	100	100	100	0	100	16.7	0
Ceftazidime (1.16, 20, 130)	*E. coli*	100	0	0	0	0	0	100	0	0
	*P. aeruginosa*	100	83.3	0	100	0	0	100	0	0
Cefoperazone-sulbactam (11/5.5, 43/21.5, 105/52.5)	*E. coli*	66.7	33.3	16.7	0	0	0	0	0	0
	*P. aeruginosa*	100	100	83.3	100	33.3	0	100	0	0
Total		88.1 (74/84)	65.5 (55/84)	57.1 (48/84)	57.1 (48/84)	45.2 (38/84)	28.6 (24/84)	61.9 (52/84)	15.5 (13/84)	4.8 (4/84)
		70.2 (177/252)			43.7 (110/252)			27.4 (69/252)		

*Data are presented as No. (%)*.

a *Antibiotic concentration (μg/mL) at T, M, P.: T, trough level; M, mid-level; P, peak level*.

For cultures treated with ampicillin, *E. faecali*s was detected in the BACTEC Plus system at all ampicillin concentrations, whereas *S. pneumoniae* only increased at T levels. The BacT/Alert FA system yielded no *E. faecalis* and *S. pneumonia* colonies at any of the ampicillin concentrations assessed. The VersaTREK system detected an increase in *E. faecali*s growth only at T levels (66.7%).

For the BACTEC plus and BacT/Alert FA systems supplemented with levofloxacin, the recovery for *S. pneumonia* was 100% at all levofloxacin concentrations. The VersaTREK system yielded *S. pneumonia* only at T levels (100%). BacT/Alert FA yielded *S. pneumonia* and *P. aeruginosa* at all levofloxacin concentrations, whereas the BACTEC plus aerobic/F and VersaTREK systems failed to recover any of the *E. coli* strains at any of the levofloxacin levels assessed. For the BACTEC plus aerobic/F and VersaTREK systems, the recovery rates for *P. aeruginosa* at T levels were 66.7 and 100%, respectively.

For cultures treated with azithromycin, the recovery rates of the BACTEC, BacT/Alert, and VersaTREK systems for *H. influenzae* were 100, 100, and 88.9%, respectively. The recovery rate of *S. pneumoniae* using the BACTEC Plus and BacT/Alert FA systems was 100% at all tested levels. For the VersaTREK system, the recovery rate of *S. pneumoniae* was 100% only at the T and M levels of azithromycin.

For cultures treated with vancomycin, recovery rates of 100% were obtained for methicillin-sensitive *S. aureus* (MSSA), methicillin-resistant *S. aureus* (MRSA), and *E. faecalis* using the BACTEC plus system. In contrast, recovery rates of 0% were obtained for MSSA and MRSA using the BacT/Alert FA and VersaTREK systems, whereas those for *E. faecalis* were 66.7 and 33.3%, respectively.

For cultures treated with ceftazidine, the recovery rates of *E. coli* using the BACTEC, BacT/Alert, and VersaTREK systems were 33.3, 0, and 33.3%, respectively, whereas those for *P. aeruginosa* were 61.1, 33.3, and 33.3%, respectively.

For cultures treated with cefoperazone/sulbactam, the recovery rates of *E. coli* using the BACTEC plus, BacT/Alert, and VersaTREK systems were 38.9, 0, and 0%, respectively, whereas those for *P. aeruginosa* were 94.4, 44.4, and 33.3%, respectively.

For cultures treated with antifungal agents, the fungal recovery rates at the T level using the BACTEC plus, BacT/Alert FA, and VersaTREK systems were 97.8% (88/90), 100% (90/90), and 78.9% (71/90), respectively, whereas the corresponding values at the P level were 90% (81/90), 96.7% (87/90), and 60% (54/90). Overall recoveries were 93.9, 98.3, and 69.4%, respectively (Table 4). The results obtained in the presented study regarding the comparison between BACTEC plus and VersaTREK culture systems are consistent with those reported previously (Riedel et al., 2011). We found that the overall recovery rate using BacT/Alert FA was higher than that of the other two systems (*P* < 0.05). Although the BacT/Alert FA system has higher recovery rates than the BACTEC plus system at the T and P concentrations, the differences were non-significant (*P* = 0.497 at the T level and *P* = 0.073 at the P level). Furthermore, with the exception of caspofungin, the BACTEC and BacT/Alert systems displayed similar recovery rates for cultures treated with antifungal drugs. For cultures treated with caspofungin, the recovery rates of *C. albicans* using the BACTEC plus and BacT/Alert systems were 50 and 75%, respectively, whereas those for *C. tropicalis* were 58.3 and 100% and those for *C. parapsilosis* were both 100%. All *Candida* spp. were detected using the BACTEC and BacT/Alert FA systems at all concentrations of voriconazole, fluconazole, amphotericin B, and itraconazole.

**Table 4:** *Candida* spp. recovery rate at different antifungal agent concentrations.

**Drug (Concn)^a^**	**Organism**	**No. (%) of bottles positive at indicated concentration**					
		**BACTEC PLUS Aerobic/F**		**BacT/Alert FA**		**REDOX 1**	
		**T**	**P**	**T**	**P**	**T**	**P**
Voriconazole (3.06, 4.7)	*C. albicans*	100	100	100	100	100	100
	*C. tropical*	100	100	100	100	100	100
	*C. parapsilosis*	100	100	100	100	0	0
Fluconazole (4.18, 6.72)	*C. albicans*	100	100	100	100	100	100
	*C. tropical*	100	100	100	100	100	100
	*C. parapsilosis*	100	100	100	100	100	100
Amphotericin B (0.5, 2)	*C. albicans*	100	100	100	100	100	0
	*C. tropical*	100	100	100	100	100	100
	*C. parapsilosis*	100	100	100	100	100	100
Caspofungin (1.6, 8)	*C. albicans*	100	0	100	50	0	0
	*C. tropical*	66.7	50	100	100	0	0
	*C. parapsilosis*	100	100	100	100	100	0
Itraconazole (0.523, 3.021)	*C. albicans*	100	100	100	100	100	100
	*C. tropical*	100	100	100	100	100	100
	*C. parapsilosis*	100	100	100	100	100	0
Total		97.8 (88/90)	90 (81/90)	100 (90/90)	96.7 (87/90)	78.9 (71/90)	60 (54/90)
		93.9 (169/180)		98.3 (177/180)		69.4 (125/180)	

*Data are presented as No. (%)*.

a *Antibiotic concentration (μg/mL) at T, P.: T, trough level; P, peak level*.

For cultures treated with voriconazole, the recovery rate of *C. parapsilosis* using the VersaTREK REDOX 1 system was 0% at trough and peak voriconazole concentrations. For cultures treated with amphotericin B, an increase in the recovery rate of *C. albicans* was obtained only at T levels using the VersaTREK system. For cultures treated with caspofungin, the recovery rates for *C. albicans* and *C. tropicalis* using the VersaTREK system were 0% at all tested concentrations, whereas that of *C. parapsilosis* was 100% at the T level. For cultures treated with itraconazole using the VersaTREK system, the recovery rate for *C. parapsilosis* was 0% at the P level and 100% at the T level, whereas that for both *C. albicans*, and *C. tropicalis* was 100% at trough and peak concentrations.

As shown in Table 5, at 0 h, the BacT/Alert system rapidly adsorbed the antifungal drugs at high concentrations, and the residual antifungal drug levels detected using the BACTEC, BacT/Alert, and VersaTREK systems were 71.7, 13, and 95.7%, respectively, whereas at T levels, the residual levels were 72.4, 11.8, and 94%, respectively. After 1 h, the levels of residual antifungal agents using the BACTEC, BacT/Alert, and VersaTREK systems were 8.4, 3.1, and 95.7%, respectively, whereas at T levels, the residual levels were 5.7, 2.2, and 88.2%, respectively. We found that when using the BacT/Alert system, the residual rate for voriconazole at 0 h was significantly lower than that detected using the other two systems, and at all concentration. (*P* < 0.05). At 1 h, the residual rate for voriconazole using the BacT/Alert system was significantly lower than that when using the other two systems at the peak concentration, although was comparable with that of the BACTEC system at the trough concentration (*P* = 0.079).

**Table 5:** Mean concentrations and percentage of remaining itraconazole after 0 and 1 h incubation in different bottles.

	**0 h**		**1 h**	
	**P**	**T**	**P**	**T**
BACTEC PLUS	0.963 (71.7)	0.633 (72.4)	0.113 (8.4)	0.050 (5.7)
BacT/Alert FA	0.153 (13.0)	0.090 (11.8)	0.037 (3.1)	0.017 (2.2)
REDOX 1	0.500 (95.7)	0.320 (94.0)	0.500 (95.7)	0.300 (88.2)

*Data are present as μg/mL (%). T, trough level; P, peak level*.

**Figure 1:**
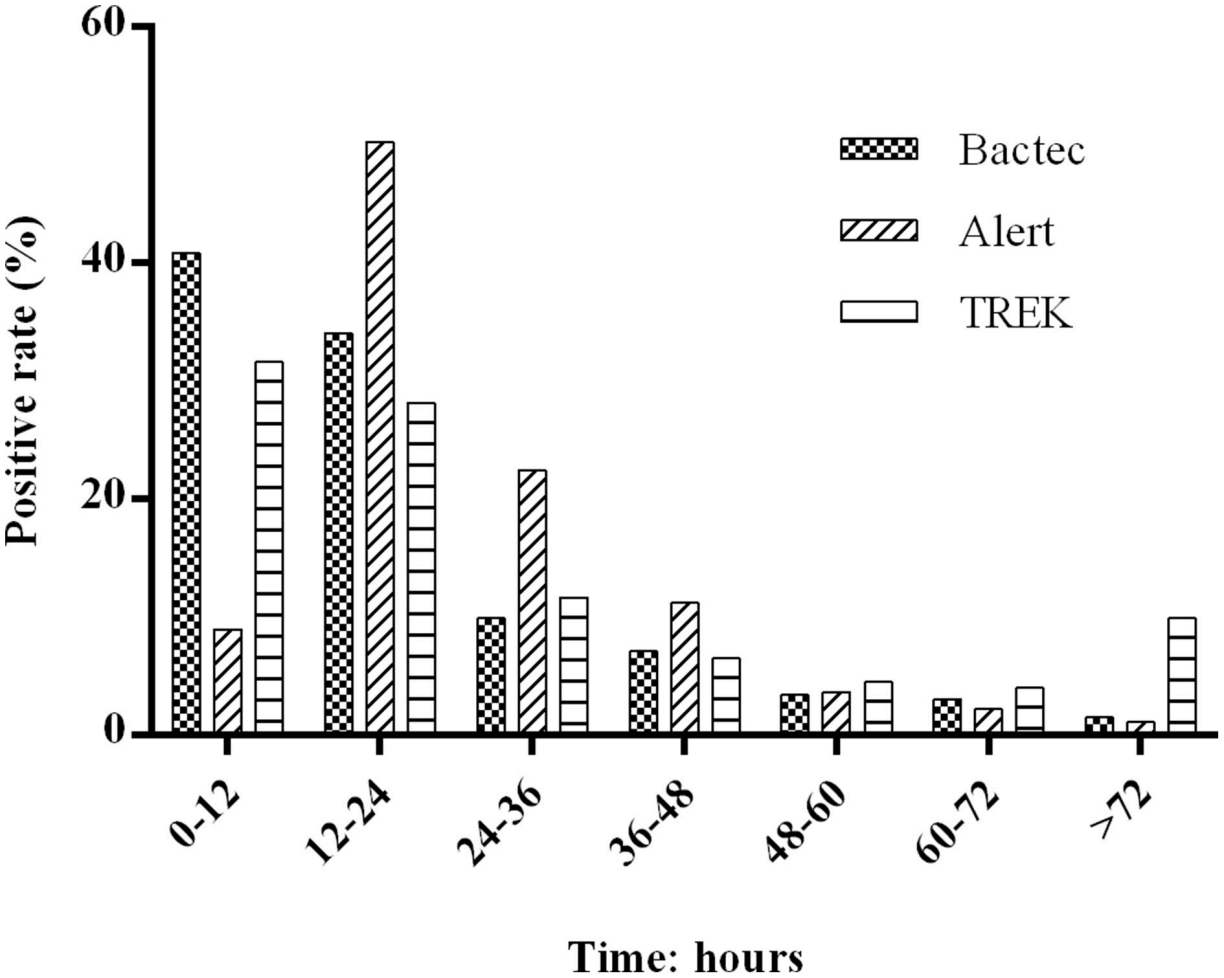
Distribution of positive blood cultures according to their time to detection during the 72 h after collection. Totals of 3,689, 4,021, and 4,427 bottles of positive cultures was recorded using the BACTEC, BacT/Alert, and VersaTREK system, respectively. The BACTEC system comprises BACTEC PLUS Aerobic/F and BACTEC lytic/10 Anaerobic/F; the BacT/Alert system comprises BacT/Alert FA /FN; and the VersaTREK systems comprises REDOX 1 and REDOX2. The percentage of positive cultures for each 12-h period represents the ratio of positive bottles to all positive bottles during this time.

Using the BACTEC, BacT/Alert, and VersaTREK systems, we recorded the TTDs of positive cultures in 3,689, 4,021, and 4,427 bottles, respectively. For the BACTEC and VersaTREK systems, we observed initial detection peaks at incubation times between 0 and 12 h for 40.1 and 31.6% of samples, respectively. For the BacT/Alert system, we recorded TTD between 0 and 12 h in only 8.9% of bottles, and for 50.3% of samples, the first peak was observed between 12 and 24 h. The number of flags accounted for 98.5, 98.9, and 90.1% of all positive specimens within 72 h (Figure 1).

**Figure 2:**
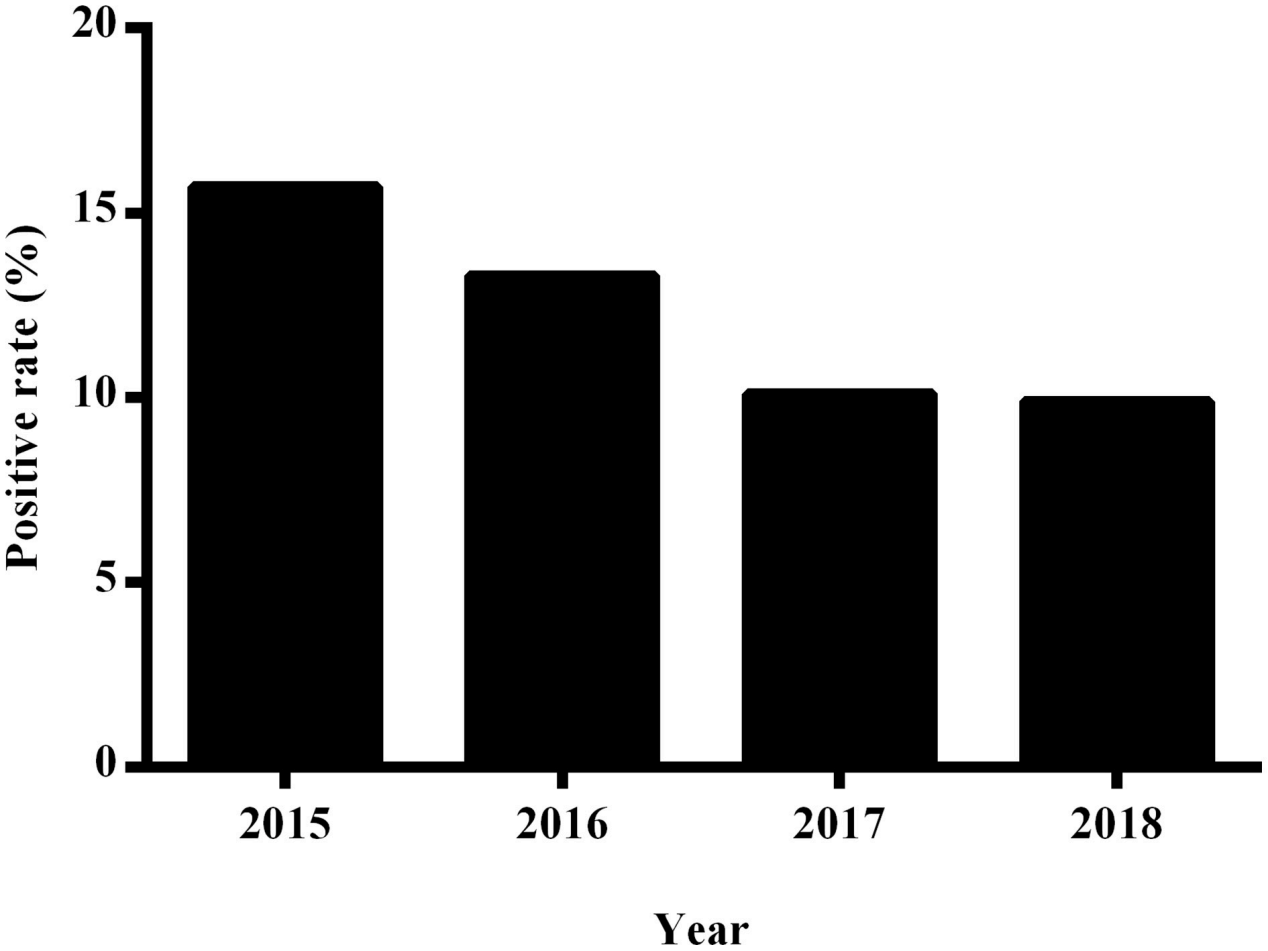
The annual positive rate of blood cultures at the First Affiliated Hospital of Zhengzhou University from 2015 to 2018.

During the period from 2015 to 2018, we detected a downward trend in the percentage of positive blood cultures derived from clinical specimens, with positives rates of 15.7% (1,597 of 8,577 patients), 13.3% (1,888 of 12,290 patients), 10.1% (1,416 of 12,853 patients), and 9.9% (1,648 of 15,003 patients) being recorded for the years 2015, 2016, 2017, and 2018, respectively. Moreover, we detected significant difference among the percentages of positive cultures obtained using the three blood culture systems (*P* < 0.001; Figure 2).

We analyzed the sensitivity of members of the family *Enterobacteriaceae* to commonly used drugs, including cefepime, piperacillin-tazobactam, aztreonam, ceftazidime, levofloxacin, and amikacin, among 735, 816, and 1,540 strains isolated between 2015 and 2018 using the BACTEC, BacT/Alert, and VersaTREK systems, respectively, In a general ward (in which virtually no antibiotics were used prior to obtaining samples for blood culture), the sensitivity to antibiotics remained stable. The *Enterobacteriaceae* strains isolated from an ICU (in which antibiotics were used in more than 90% of instances prior to blood culture) using the BACTEC and BacT/Alert systems showed similar sensitivities, whereas, with the exceptions of amikacin and levofloxacin, the sensitivities of strains cultured using the VersaTREK system were significantly reduced (*P* < 0.05) (Table 6).

**Table 6:** Comparison of the antibiotic sensitivities of *Enterobacteriaceae* isolated using three blood culture systems (%).

**Antibiotic**	**General ward**				**ICUs**			
	**BACTEC^a^*n* = 537**	**BacT/Alert^a^*n* = 622**	**TREK^a^ 2017 *n* = 588**	**TREK^a^ 2018 *n* = 683**	**Bactec^a^*n* = 196**	**BacT/Alert^a^*n* = 201**	**TREK^a^ 2017 *n* = 200**	**TREK^a^ 2018 *n* = 327**
Cefepime	65.4	63.3	66.8	63.2	65.9	63.3	54	44.3
Piperacillin-tazobactam	86	83.7	84.1	81.5	73	74.1	58.3	48.3
Aztreonam	56.1	56.2	59.5	55.7	48.5	51	36.7	31.9
Ceftazidime	63.2	62.8	67.1	65.5	54.9	53.7	44.5	38.6
Levofloxacin	44.5	44.8	43.9	44.6	41.8	45.3	37.2	35.2
Amikacin	91.4	91.3	92.7	94.2	83.7	85.5	82	81.5

*Data are presented as No. (%). n represents the number of strains isolated from the corresponding blood culture system*.

a *Strains were isolated using the BACTEC system in 2015*.

b *Strains were isolated using the BacT/Alert system in 2016*.

c *Strains were isolated using the VersaTREK system in 2017*.

d *Strains were isolated using the VersaTREK system in 2018*.

## Discussion

Numerous studies have compared the three blood culture systems assessed in the present study for the recovery of common clinical pathogens (Horvath et al., 2003, 2004, 2007; Søgaard et al., 2006; Flayhart et al., 2007; Mirrett et al., 2007; Miller et al., 2011; Riedel et al., 2011). In the present study, we performed a simulation study using a larger range of pathogens and antibiotics or antifungals to compare the differences in TTD and recovery among the three blood culture systems. Clinical data obtained from January 2015 to December 2018 using the three blood culture systems were used for comparative analyses. The performances of the three blood culture systems and their effect on clinical treatment were further investigated.

Using the three blood culture systems, we compared the TTD of pathogens in the absence of antibiotics. For aerobic cultures, the VersaTREK REDOX 1 system displayed the shortest TTD for six of the 12 pathogens assessed, and for five pathogens the TTD values obtained were comparable to those obtained using the BACTEC PLUS Aerobic/F culture system. The BacT/Alert FA system, however, did not show any advantages with regards to detecting aerobic cultures.

For anaerobic cultures, the BACTEC lytic/10 anaerobic/F system containing hemolysin displayed the shortest TTD for six of 10 pathogens. However, despite a lack of mechanical agitation, the VersaTREK REDOX2 system was unable to generate an anaerobic environment, and hence was unsuitable for culturing anaerobic organisms. Comparatively, the BacT/Alert FN system showed no discernible advantage.

On the basis of our comparison of anaerobic cultures, the status of *H. influenzae* growing in BacT/Alert FN bottles can effectively reflect the ability of this system to detect fastidious bacteria. *P. aeruginosa* grew only in the VersaTREK REDOX 2 system, probably owing to the fact that the VersaTREK system is exposed to the external environment via the connector. Thus, the upper space inside the bottle was not completely anaerobic and an anaerobic environment was maintained only at the bottom the bottle. In all three of the culture systems assessed, *B. melitensis* did not alarm in the three anaerobic blood culture flasks within 5 days. Since most clinically isolated pathogens are either aerobic or facultatively anaerobic bacteria, the aeration produced by mechanical agitation of the VersaTREK system contributes to the excellent performance of the REDOX1 system.

Numerous studies have indicated the importance of eliminating antibiotic and antifungal agents from blood cultures of potential pathogens (Spaargaren et al., 1998; Ziegler et al., 1998; Flayhart et al., 2007; Miller et al., 2011; Riedel et al., 2011; Roh et al., 2012; Sullivan et al., 2013; Zadroga et al., 2013). The findings of the present study show that the ability of resins to adsorb antibiotics and antifungal drugs facilitates the rapid elimination of antibiotics and antifungal drugs at a certain plasma concentration, thereby shortening the TTD and increasing the positive rate of detection. The resins used in blood culture systems exhibit excellent adsorption capacity for vancomycin, showing a high detection rate at the highest serum concentration. In addition, in the present study, we compared systems for their ability to eliminate macrolides, due to the lower serum concentration of azithromycin, and found that both the adsorption and dilution methods can effectively eliminate the effects of these antibiotics. However, compared with activated carbon, resins show less efficient quinolone absorption, resulting in a higher detection rate than that for levofloxacin when using the BacT/Alert FA system. On the basis of our comparison of antifungal drugs, we found that cultures containing activated carbon displayed a better performance, particularly with respect to caspofungin. In samples supplemented with voriconazole, activated carbon resulted in significant adsorption within a short period (immediately). In terms of performance, we found the VersaTREK system to be unsatisfactory, as it was necessary to increase blood volume in order to enhance pathogen detection, which concomitantly resulted in an increase in antibiotic levels.

We retrospectively analyzed the TTD of the three blood culture systems using clinical specimens. Previous studies have reported that a combination of the BACTEC PLUS Aerobic/F and BACTEC lytic/10 anaerobic/F systems contributes to higher positive rates and a shorter TTD (Almuhayawi et al., 2015; Rocchetti et al., 2016), which is consistent with the findings of our simulated studies and clinical data. After 24 h in the machine, the detection rates of the BACTEC, BacT/Alert, and VersaTREK systems were 74, 58, and 59%, respectively, and we found that the BACTEC system had a shorter TTD than the other two systems.

The BacT/Alert system has been produced with bottles containing resins instead of activated carbon, and studies that have been performed to compare the performance of these two media indicate that the adsorption of antibiotics by resins is higher than that of activated carbon, with an increased recovery rate and shorter TTD (Mitteregger et al., 2013; Doern et al., 2014; Fiori et al., 2014; Kirn et al., 2014). However, in terms the positive detection rate within 12 h of culture, resins were found to have no advantage over the standard bottles containing activated carbon (Amarsy-Guerle et al., 2015). Fiori et al. demonstrated that the BACTEC system showed a better recovery rate for gram-negative bacteria, whereas the BacT/Alert resin bottles showed a higher detection rate for gram-positive bacteria (Fiori et al., 2014). In contrast, for anaerobic organisms, the performance of resins was comparable with that of activated carbon, thus reducing the willingness of users to change media (De Keukeleire et al., 2015; Mueller-Premru et al., 2017).

Among the strains isolated from positive blood cultures, the proportion of bacteria in the family *Enterobacteriaceae* annually exceeds 50% (data not shown). We believe that a certain concentration of antibiotics in the blood will directly affect pathogen detection, particularly for sensitive bacteria, potentially yielding false-negative results, and in this respect, our simulated study revealed differences in the ability of the three assessed blood culture systems to eliminate antibiotics.

We retrospectively analyzed the sensitivities of *Enterobacteriaceae* isolated using the three systems, and notably observed that whereas the sensitivities of strains isolated from a general ward were similar, those of the strains isolated from an ICU were relatively dissimilar. Furthermore, we found that, although the sensitivities of *Enterobacteriaceae* isolated using the BACTEC and BacT/Alert systems were similar, the recovery rate using the BACTEC system in the simulated study was high at peak and medium concentrations, whereas most blood cultures are carried out at lower concentrations. The sensitivities of *Enterobacteriaceae* isolated using the VersaTREK system were, however, found to be markedly different from those observed using the other two systems, notably with respect to an increase in the proportion of multi-drug resistant bacteria. Nevertheless, such an outcome may not accurately reflect the prevalence of bacterial resistance in hospitals, thus leading to the use of higher antibiotic concentrations in empirical medicine.

The major limitation of this study is that the clinical data are of a before–after type and a direct comparison among the different systems was not feasible. However, our simulated study and the clinical data have been mutually confirmed. Moreover, there were no changes in antibiotic usage or medical activities at the study hospital during the four consecutive years for which data were obtained.

In conclusion, this study shows that a combination of the BACTEC PLUS Aerobic/F and BACTEC lytic/10 anaerobic/F systems displayed the best clinical performance. On the basis of our findings, we recommend that, in China, the commercially available BacT/Alert FAN system should potentially be considered for culturing clinical blood specimens, whereas the VersaTREK REDOX system would be more suitable for primary culture of blood samples lacking antibiotics.

## Data Availability

All datasets generated for this study are included in the manuscript/supplementary files.

## Author Contributions

GL, JS, and LM conceived and designed the work. SP, SZ, WL, and HX performed the study. GL and XS analyzed the data. GL and YW wrote the manuscript. All authors read and approved the final manuscript.

### Conflict of Interest Statement

The authors declare that the research was conducted in the absence of any commercial or financial relationships that could be construed as a potential conflict of interest.
